# Dose-Dependent Physiological Response to Transient Bioaccumulation of Tetracycline in Kimchi Cabbage (*Brassica campestris* L.)

**DOI:** 10.3390/antibiotics14050501

**Published:** 2025-05-13

**Authors:** Hadjer Chohra, Keum-Ah Lee, Hyeonji Choe, Ju Young Cho, Vimalraj Kantharaj, Mi Sun Cheong, Young-Nam Kim, Yong Bok Lee

**Affiliations:** 1 Division of Applied Life Science (BK21 Four), Gyeongsang National University, Jinju 52828, Republic of Korea; chohra.hadjer@gnu.ac.kr (H.C.);; 2 Institute of Agriculture and Life Science (IALS), Gyeongsang National University, Jinju 52828, Republic of Korea; lka830815@gmail.com (K.-A.L.);; 3 Department of Crop Science, Kyungpook National University, Sangju 37224, Republic of Korea

**Keywords:** veterinary antibiotic, phytotoxicity, tetracycline accumulation, crop performance, food safety

## Abstract

Background/Objectives: Globally, antibiotic contamination has become an emerging issue in agricultural lands. The presence of antibiotic residues in farmlands, especially through the application of manure fertilizers containing veterinary antibiotics, e.g., tetracycline (TC), can cause severe toxicity, which inhibits crop growth and performance, subsequently threatening human health via consumption of contaminated products. This study was conducted to evaluate the phytotoxicity of TC on Kimchi cabbage (*Brassica campestris* L.) during seed germination, seedling, and vegetative growth stages, along with its physiological responses and bioaccumulation under TC stress. Methods: The responses of cabbage plants to TC stress were assessed through a germination test and a pot experiment, conducted for three days and six weeks, respectively, under different doses of TC (0, 5, 10, 25, and 50 mg/L). Results: As a result of the germination test, higher TC doses (25 and 50 mg/L) tended to delay seed germination, but all treatments achieved a 100% germination percentage by Day 3 after sowing. Eight days after sowing, the length of shoots and roots of seedlings exhibited a TC dose-dependent decline, specifically under 50 mg TC/L, showing a considerable decrease of 24% and 77%, respectively, compared to control. Similar results were observed in the plants transitioning from the seedling to vegetative stages in the pot experiment. Four and six weeks after sowing, the 50 mg TC/L dose showed the strongest phytotoxicity in cabbage plants with physiological parameters, such as the maximum photosystem II quantum yield (*F*_v_/*F*_m_), pigment content (chlorophyll and carotenoid), biomass, and leaf number, significantly reduced by 26 to 60% compared to control. Interestingly, at lower TC doses (5 and 10 mg/L), a hormesis effect was observed in the phenotype and biomass of the plants. In addition, the degree of TC accumulation in the plants was highly dose-dependent at Week 4 and Week 6, but a temporal decline in TC accumulation was noted between these time points in all TC treatments. This phenomenon might affect the value of the bio-concentration factor (BCF) as an indicator of the plant’s tendency to uptake TC. That is, in Week 6, the dose-dependent reduction in BCF for TC in the plants was likely attributed to a dilution effect caused by plant biomass increase or a degradation mechanism within the plant. Conclusions: Overall, our findings suggest that tetracycline toxicity induces seed germination delay and influences seedling elongation and photosynthetic functions, ultimately impairing crop growth and performance. Also, the antibiotic dynamics related to accumulation and degradation in plants were identified. These results will not only suggest the toxicity threshold of TC for cabbage but also provide insights into effective soil management strategies for food production safety and agroecosystem sustainability in antibiotic-contaminated soils.

## 1. Introduction

Since their introduction in the 1940s, antibiotics have played a groundbreaking role in treating and preventing infectious diseases worldwide. Over the past several decades, this has led to significant economic benefits, including reduced healthcare costs and increased production of meat and dairy [1,2]. Although antibiotic usage varies greatly across countries, more than 70% of the world’s total antibiotic consumption is attributed to livestock [3]. As of 2018, data reported from 109 countries in the World Organization for Animal Health region indicated that the annual consumption of veterinary antibiotics (VAs) was approximately 76,700 tons [4]. Furthermore, global consumption of VAs is projected to rise by 11.5% between 2017 and 2030 [5]. Among the VAs, tetracyclines and penicillins are the most widely used antimicrobial agents in animal healthcare, accounting for 40.5% and 14.1% of the total consumption, respectively [4]. However, in recent years, the overuse of VAs has become widespread, leading to their classification as emerging contaminants in various environments [3,6]. Despite this, no strict safety regulations have been established to address the issue.

Globally, antibiotics are present in various ecosystems, which is attributed principally to the transition and release of VAs being excreted undigested from digestive tracts (approximately 75% animal intake) to the aquatic (surface water, groundwater, sea, etc.) and terrestrial (sediment and soil) environments [5,7,8,9]. In agricultural ecosystems, VAs in various concentration ranges have been detected and this is mainly considered a legacy of livestock manure application and wastewater irrigation. According to previous studies reviewed by Yang et al. [9], the antibiotics were detected in various concentrations ranging from trace levels to hundred µg/g and several hundred ng/L to a few ten µg/L in manures and wastewater, respectively. Therefore, the input of such VA-contaminated mediators into the soil environment can trigger antibiotic contamination in agricultural soil, eventually degrading soil quality and crop productivity [10]. Moreover, the extent of antibiotic residues in farmland soils can vary with the source of manures. According to Wei et al. [11], the highest concentrations were found in soils treated with poultry, swine, and cow manures, in that order. Additionally, the levels of antibiotic contaminants differed with soil depth due to their absorption, migration, and stability throughout the soil profile [12].

Tetracyclines, including tetracycline (TC), chlortetracycline, oxytetracycline, etc., are major antibiotics that comprise ca. 66% of the total use in livestock, and have been widely used due to their wide range of benefits, including low cost and therapy for infectious diseases [12,13,14]. Additionally, these antibiotics have contributed to improving the breeding process of livestock, such as poultry and cattle, and thus increasing agricultural productivity, which is mainly related to improved feed efficiency and increased nutrient absorption [15]. As such, considering the high production and use of tetracyclines worldwide, e.g., ranked second in the world and first in China, there is no doubt that they are a major cause of antibiotic contamination of agricultural soils mainly by adding livestock manures and wastewater irrigation [16]. Minden et al. [17] reported that the TC concentration ranged from 10 to 15 µg/kg in conventional agricultural fields with manure fertilization. Due to this, currently, antibiotic-resistant bacteria are enriched in agricultural lands, and they subsequently enable their transfer to different ecological niches [18], which is one of the high potential causes threatening human health. Moreover, antibiotic residues in farmlands can induce nutrient deficiency and phytotoxicity, thereby inhibiting the growth and performance of crops [18,19,20]. It was reported that the phytotoxic effects of TC in agricultural land have been observed in edible crops, including tomato (*Solanum lycopersicum* L.), cucumber (*Cucumis sativa* L.), and lettuce (*Lactuca sativa* L.) [21]. These crops absorbed TC from their roots, then accumulated the antibiotic in their edible parts and altered its metabolism within the plant tissue ultrastructure. As such, in agroecosystems, the emerging antibiotic contamination through the continuous addition of VAs via manure application and wastewater irrigation would affect the overall quality and productivity of agricultural soils. However, the toxicity of VAs may vary depending on crop species and soil management approaches [22,23]. Thus, more studies are needed on crop stress screening for antibiotics, including tetracycline.

Chinese cabbage (*Brassica campestris* L. ssp. *pekinensis*), also called Kimchi cabbage, is one of the vegetables with the highest demand in eastern Asia. It is known that cabbage species contain high nutrients (e.g., Ca, K, Fe, etc.) and metabolites (e.g., glucosinolates, flavonoids, carotenoids, phenolic acids, vitamin C, folic acid, carbohydrates, amino acids, etc.) that are beneficial for human health [24,25]. As of 2022, approximately 2.2 million tons of cabbages were produced, accounting for 26.3% of Korean vegetable consumption, as reported by the Rural Development Administration [26]. In general, livestock manures are widely applied as a basic fertilizer in uplands for the cultivation of crops, including cabbage, so it can be presumed that the introduction of manure-derived VAs will ultimately affect the growth and productivity of crops [27]. Additionally, the continuous entry of VAs into the soil environment allows them to become pseudo-persistent contaminants affecting nontarget organisms [28]. However, studies on the toxic effects of antibiotics on vegetables grown in soil substrate conditions are lacking. This study was performed to investigate the germination, growth, and physiological responses of *B. campestris* to contaminated soils treated with different doses of tetracycline. To minimize the impacts of edaphic and climate variations on TC toxicity, transport, and accumulation, all plants were cultivated in artificial soil and grown under controlled conditions, such as temperature and relative humidity.

## 2. Results

### 2.1. Germination and Early Seedling Growth

The germination percentages of cabbage seeds differed depending on the TC dose one day after sowing. The highest GPs were observed in control (ca. 92%), followed by 5, 10, 25, and 50 mg/L of TC doses. Compared to control, GPs in 25 and 50 mg TC/L were significantly lower (17% and 22%, respectively; *p* < 0.05, Table 1). All seeds in control were germinated on Day 2, while those in the remaining treatments did on Day 3.

**Figure 1:**
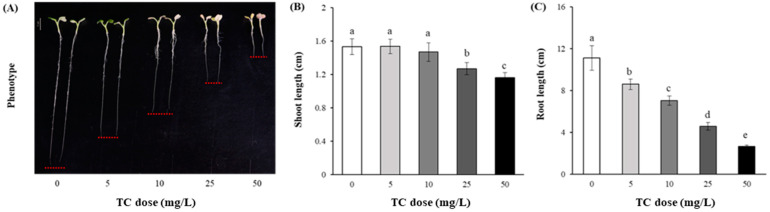
Growth of 8-day-old cabbage (*B. campestris* L.) seedlings germinated under different tetracycline (TC) doses: phenotype (**A**), shoot length (**B**), and root length (**C**). Data are mean ± standard deviations (*n* = 3). Different letters indicate significant differences among the TC treatments (Tukey’s HSD, *p* < 0.05).

Eight days after sowing, the growth of the cabbage seedlings showed a considerable difference in their phenotype among the TC treatments (Figure 1A). The highest length of the seedling shoots was observed in control and 0.5 mg TC/L treatment (1.53 cm), followed by 10, 25, and 50 mg TC/L treatments (1.47, 1.27, and 1.16 cm, respectively; Figure 1B). A more significant difference was found in the root length among the TC treatments (*p* < 0.05), and it steadily decreased as the TC dose increased (Figure 1C). The root lengths in 5, 10, 25, and 50 mg TC/L treatments were significantly lower (22% to 77%) than that in control (*p* < 0.05).

Likewise, the seedlings’ FW represented a decreasing trend as the TC dose increased: 0 (29.4 mg) > 5 mg/L (25.7 mg) > 10 mg/L (25.1 mg) > 25 mg/L (21.8 mg) > 50 mg/L (16.7 mg).

### 2.2. Growth and Physiological Responses at the Vegetative Stage

#### 2.2.1. Plant Growth and Development

**Figure 2:**
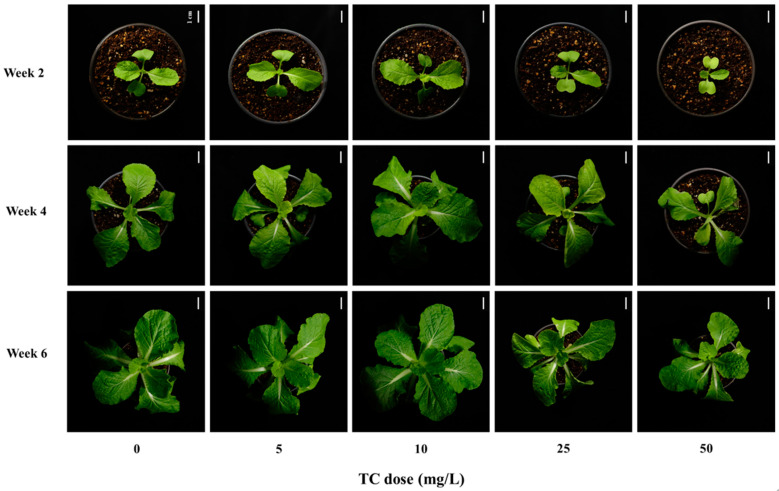
Morphological changes in cabbage (*B. campestris* L.) seedlings treated with different tetracycline doses for 6 weeks. The white bar in the upper right corner of each photo indicates a size of 1 cm.

The phenotype of cabbage plants biweekly was recorded as shown in Figure 2. During the cultivation, inhibition of the plant growth caused by TC exposure was observed in 25 and 50 mg/L, while, compared to control, the TC doses of 5 and 10 mg/L appeared to induce hormesis effect on the plant phenotype, particularly in Week 4 (Figure 2).

**Figure 3:**
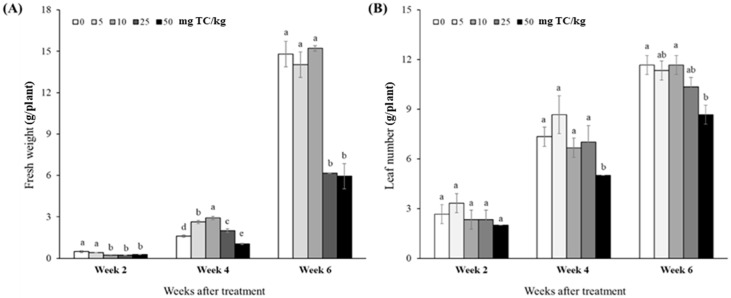
Growth parameters of cabbage (*B. campestris* L.) seedlings treated with different tetracycline (TC) doses: fresh weight (**A**) and leaf number (**B**) measured bi-weekly after treatment. Data are mean ± standard deviations (*n* = 3). Different letters indicate significant differences among the TC treatments (Tukey’s HSD, *p* < 0.05).

Likewise, the fresh weight (FW) of the cabbage plants differed with TC dose level and temporal change (Figure 3A). In Week 2, the FW values in control and 5 mg TC/L were significantly higher than those in the remaining TC treatments (*p* < 0.05). In Week 4, the doses of 5 to 25 mg TC/L had considerably higher FW values (2.00 to 2.93 g) compared to the control (1.61 g; *p* < 0.05), while the lowest FW value was found in 50 mg TC/L (1.05 g), which was significantly lower than that of control (*p* < 0.05). In Week 6, the FW of the control plant attained 14.8 g, which was similar to the plant FW values in the TC doses of 5 and 10 mg/L (*p* > 0.05). Meanwhile, the marked reduction in the plants’ FW was observed in the TC doses of 25 and 50 mg/L (58.3% and 59.8%, respectively), compared to control (*p* < 0.05). Moreover, the leaf number (LN) of plant leaves tended to be negatively affected by TC treatment (Figure 3B). Among the TC doses, the highest level of 50 mg/L had significantly lower LN in Week 4 and Week 6 (31.8% and 25.7%, respectively), compared to control treatments (*p* < 0.05).

Overall, the growth response of kimchi cabbage to various concentrations of TC appeared to be toxic at concentrations above 25 mg TC/L; however, this toxicity diminished during the maturation process at concentrations below that.

#### 2.2.2. Photosynthetic Performance and Pigmentation

There was no significant difference in the maximal efficiency of photosystem II (PS II) photochemistry (*F*_v_/*F*_m_) of cabbage leaves among the TC dose treatments in Week 2 (*p* > 0.05; Table 2). On the other hand, in Week 4, the lowest value was observed in 50 mg TC/L treatment, significantly lower (36.9%) than that in control (*p* < 0.05). Similarly, in Week 6, the *F*_v_/*F*_m_ value was also lowest in 50 mg TC/L, but not significantly different compared to the control (*p* > 0.05).

The SPAD values of cabbage leaves (19.2 to 21.1) measured in Week 2 showed no significant difference among the TC dose treatments (*p* > 0.05). Meanwhile, the photosynthetic pigment content in the leaves tended to differ considerably with the TC doses in the other measurement times (Table 2). In Week 4, both chlorophyll and carotenoid contents were highest in control (356 and 50 mg/g, respectively), intermediate in 5, 10, and 25 mg TC/L (209–261 and 27–36 mg/g, respectively), and lowest in 50 mg TC/L treatment (198 and 25 mg/g, respectively). Similarly, in Week 6, the highest values of these pigments were found in control (356 and 56 mg/g, respectively), but the lowest values were found in 25 mg TC/L treatment (211 and 36 mg/g, respectively).

#### 2.2.3. Tetracycline Concentrations in Soil and Plant

**Figure 4:**
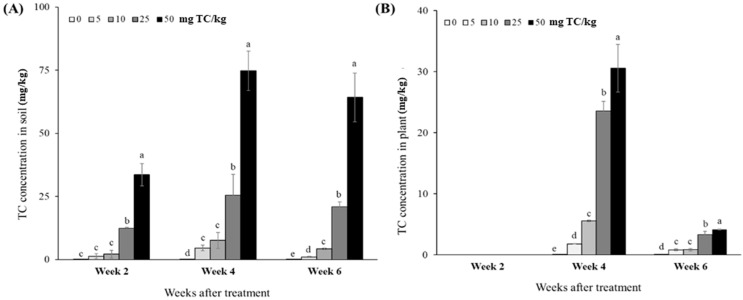
Concentrations of tetracycline (TC) in soils (**A**) treated with different TC doses and those in seedlings of *B. campestris* L. (**B**), measured bi-weekly after treatment. Data are mean ± standard deviations (*n* = 3). Different letters indicate significant differences among the TC treatments (Tukey’s HSD, *p* < 0.05).

The concentration of TC in soil was highly dependent upon TC doses (Figure 4A). In other words, the TC accumulated in the soil increased as the TC doses increased. Two weeks after the initial TC treatment, the TC concentrations in the soils of all treatments were greatly lower than intended in this study. On the other hand, one week after the second TC treatment, the concentrations of TC in the soils appeared close to the intended levels. However, two weeks later, the TC concentrations in the soils slightly decreased in Week 6.

While no data for TC accumulated in cabbage plants in Week 2 were shown due to a lack of sample amount, the concentration of TC in the plants in Week 4 was detected in proportion to the TC doses treated in the soils (Figure 4B). The highest TC concentration was observed in 50 mg TC/L (30.6 mg/kg), followed by 25, 10, 5, and 0 mg TC/L (23.6, 5.6, 1.8, and 0 mg/kg, respectively) treatments. On the other hand, in Week 6, much lower TC concentrations (54–89%) were found in each treatment than in Week 4. The TC dose of 50 mg/L had the highest TC concentration (4.2 mg/kg) in the plants, followed by 25, 10, 5, and 0 mg/L (3.3, 0.9, 0.8, and 0 mg/kg, respectively).

#### 2.2.4. Bioconcentration Factor and Accumulation

Concerning the TC absorption capacity of cabbage plants, the ratio of the bio-concentration factor (BCF) in Week 4 was highest (0.780) in 25 mg TC/L, which was significantly higher (1.7–1.9 times) than that in 5 and 50 mg TC/L (Table 3). Meanwhile, in Week 6, a low level of TC (5 mg/L) exhibited the highest BCF value (0.636) significantly higher than the remaining TC treatments (0.066–0.213; *p* < 0.05). For the total amount accumulated in the plants, the highest values were observed in 50 mg TC/L in both sampling times, and then the accumulated amount decreased as the TC dose was lowered.

#### 2.2.5. PCA Analysis

**Figure 5:**
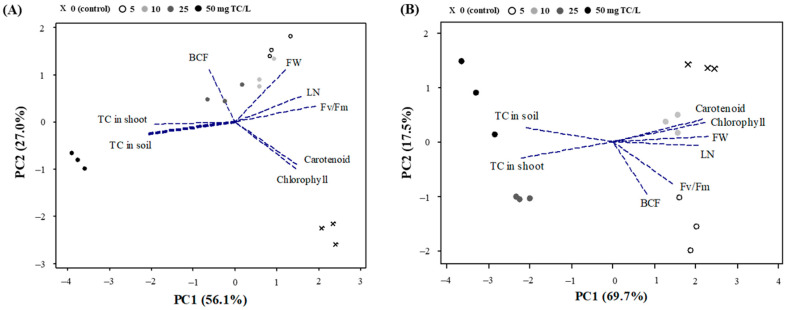
Biplot graph of principal component analyses (PCA) of the variables, including fresh weight (FW), leaf number (LN), photosynthesis fluorescence (*F*_v_/*F*_m_), chlorophylls, carotenoid, concentrations of tetracycline (TC) in soil and shoot, and bioconcentration factor (BCF), of *B. campestris* seedlings grown under different TC doses for 6 weeks: data for Week 4 (**A**) and Week 6 (**B**).

Multivariate analysis of all parameters determined in this study separated each TC dose clearly on the two PCA axes (Figure 5). In Week 4 (Figure 5A), the treatments with different TC doses were split principally due to *F*_v_/*F*_m_, LN, carotenoid, chlorophyll, and TC in shoot and soil along the first PC1 axis (56.1% variance, *p* < 0.01). On the other hand, along the PC2 axis (27.0% variance), the TC treatments were separated mainly with BCF, FW, carotenoid, and chlorophyll (*p* < 0.05, Figure 5A). In Week 6, along the PC1 axis explaining 61.8% of the total variations, the TC treatments were separated by FW, chlorophyll, carotenoid, LN, and TC concentrations in shoot and soil (*p* < 0.001, Figure 5B). Meanwhile, along the PC2 axis (20.7% variance), the TC treatments were split with BCF and *F*_v_/*F*_m_ (*p* < 0.01). To interpret the overall trend, the growth and physiological responses, including FW, LN pigments, and photosynthesis, showed positive relationships with TC doses of <10 mg/L or less, while the TC doses above 25 mg/L showed a negative effect along with an increase in TC accumulation in the plant shoots.

## 3. Discussion

Globally, agricultural landscapes have become a huge reservoir of veterinary antibiotics (e.g., tetracyclines, sulfonamides, etc.) due to unintentional input of VAs through manure fertilization. Due to the residues of VAs accumulated in farmlands being stable for several years, the antibiotics can be absorbed directly by various organisms (e.g., plants, earthworms, etc.) and spread into aquatic systems (e.g., streams and rivers via runoff and groundwater via leaching), leading to degradation of agroecosystem services [18,29]. So, there is great concern that the dynamics of VA within the agroecosystem will eventually harm agricultural sustainability and threaten human health via the food chain [30]. In fact, among the pathways through which antibiotics and antibiotic-resistant bacteria are inhaled into humans, the crop absorption process has been recently studied but is still insufficient. Therefore, in this study, we investigated the response of Kimchi cabbage, Korea’s most widely consumed crop, to TC toxicity, focusing on the early growth stages from germination and seedling development, which largely determine the overall quality and yield of the crop.

In the present study, the phytotoxicity of TC was lethal during seed germination and post-germination stages of *B. campestris*, as has been reported in other studies targeting various crop species. According to Liu et al. [21], seed germination of different crop species, including sweet oat (*Cichaorium endivia*), rice (*Oryza sativa*), and cucumber (*Cucumis sativus*), was suppressed at the concentration of 0–500 mg TC/L, and their EC50 values were found below 300 mg/L. Despite the relatively lower doses of TC devoted in this study than a study of Liu et al. [21], we also found an inhibitory or delaying effect at certain concentrations (mostly >25 mg TC/L) to seed germination and post-germination physiology of *B. campestris* (i.e., sowing to 8 days after sprouting) through the Petri-dish trial (Figure 1). Furthermore, Minden et al. [17] and Luo et al. [29] reported that the seed germination of *B. napus* was considerably delayed even at the lower levels of 5 and 0.5 µg TC/L, respectively. These results indicate that crop sensitivity to TC stress may vary highly with species, cultivars, traits, or exposure duration [18,21,30]. Moreover, such an effect of TC toxicity was observed in shoot and root development after germination. In particular, root elongation appeared considerably sensitive to the increment of TC dose level (Figure 1C); the root length in 50 mg TC/L was approximately three times shorter than in control. This may be due to the limited absorption capacities of water and/or nutrients from the root system affected by TC-induced phytotoxicity [29]. In addition, as roots are the first site of the stress signal, followed by the leaves [30], the harmful effects of TC on root elongation may be triggered by inducing oxidative stress, thereby weakening the overall performance of plants, including metabolic and photosynthetic functions [31,32]. In this study, the toxicity of TC affecting seed germination and post-germination seedling growth appeared in the order of root elongation > shoot elongation > seed germination. This was in line with An et al. [33] and Cheng and Zhou [34] who revealed a higher sensitive endpoint of root and shoot elongation in response to pollutant toxicity than seed germination. Presumably, this is because while the seed coats prevent VA penetration, the seedling roots and shoots are readily affected by the VA toxicity after embryonic root emergence [35].

Likewise, in the pot trial, cabbage seedlings (i.e., 2 to 6 weeks after sowing) appeared to be significantly affected by TC stress at the vegetative stage. A closer look at this TC effect revealed that the seedling growth responses varied depending on the TC dose. Especially in Week 4 and Week 6, the inhibitory effects on the growth and physiological parameters of the seedlings, such as FW, LN, *F*_v_/*F*_m_, and pigments, were observed in the higher doses of TC (>25 mg/L). The marked decline in these parameters with higher TC doses may be attributed to the cytotoxic potential of TC and the inhibition of chloroplast and mitochondrial translation, thereby repressing the seedling’s physiological function, particularly photosynthesis [36,37]. Consistent with this, significant and dose-dependent inhibitions of photosynthesis efficiency (i.e., *F*_v_/*F*_m_) and pigmentations (i.e., chlorophyll and carotenoids) were confirmed in this study, as shown in Table 2. The decreased value of *F*_v_/*F*_m_ may be attributed to photosystem (PS) II damage due to antibiotic toxicity, followed by disturbances in the electron flow between PS I and PS II [38]. Moreover, as reported in many studies, the first noticeable symptom of antibiotic toxicity in plants is a chlorotic change due to degrading chlorophylls and impairing the chlorophyll biosynthesis pathway, leading to the promotion of photoinhibition that decreases the photosynthesis efficiency [38,39]. These toxic effects of TC were consistent with those observed in various plant species: *Pisum sativum* [40], *Lemna minor* [41], *Lupinus luteus* [42], *Spinacia oleracea* [43], *Iberis sempervirens* [44], etc. Furthermore, Migliore et al. [45] and Xie et al. [37] revealed that TC with high levels may induce genotoxicity, suppression of protein synthesis, and disorder of organelle functions, resulting in phytotoxic effects on the overall growth and performance of plants from the early stages including germination and radicle elongation.

The phytotoxic damage caused by antibiotics relies on their accumulation and translocation in the soil–plant system; however, it may vary with the type and characteristics of the soil and vegetables [39,46]. In this study, we found a significant difference in the concentration of TC in soil according to TC dose, which in turn affected the uptake of TC in *B. campestris* seedlings (Figure 4). Accordingly, a marked accumulation of TC in the cabbage was observed in higher doses of TC, i.e., more than 25 mg/L, compared to control, indicating that there is a high potential for human health risk when consuming crops accumulated with TC, as well as a decline in crop productivity and quality [16,43]. On the other hand, BCF, an indicator of TC tendency to absorb in the plant, did not show consistent results across the TC doses (Table 3). According to the multivariate analysis (i.e., PCA), BCF values showed a significant negative relationship with pigment contents, including chlorophylls and carotenoids, in Week 4 (*p* < 0.05; Figure 5A), suggesting that TC stress adversely affected the physiology of cabbage at the vegetative stage immediately after TC treatment. In contrast, in Week 6, the BCF values increased at low TC dose treatment (5 mg/L) but decreased at the remaining treatments (Table 3). The significant changes in BCF values between Week 4 (immediately after TC treatment) and Week 6 (two weeks later) were likely attributed to the dilution effect caused by increased plant biomass or the degradation process occurring within the plant.

In general, tetracycline is stable in the natural environment, so has been found at high and diverse residual levels in soils, sediments, and surface and groundwater [37,47]. In addition, due to its persistence and low water solubility, antibiotic residuals in agricultural land can be readily absorbed by crops via water transport and passive absorption [48]. Meanwhile, TC can also break down macromolecules into smaller ones through non-biodegradable (e.g., ozonation, photolysis, oxidation, etc.) and biodegradable (microbial and enzymatic activations) pathways in soil and water environments. However, the degree of degradation is variable with several edaphic factors, such as pH, redox potential, and temperature [49]. Moreover, the threshold level of TC is mainly 100 µg/kg [50], easily taken up to the vegetables and crops, threatening human health as well as crop production. Indeed, the residue of TC that potentially remains in agricultural soil for several days or longer periods could cause a significant biological threat by facilitating antimicrobial resistance (AMR), which may be an important pathway for AMR and ultimately pose a serious challenge to the security of human health [51].

For plants, once antibiotics are accumulated in their organs, phytometabolization including enzymatic degradation or transformation (via hydrolysis, reduction, or oxidation) detoxifies the antibiotics and leads to the production of primary metabolites [50]. For this, plants have several enzyme candidates that facilitate degrading tetracyclines, e.g., laccases, lignin peroxidases, manganese peroxidases, and oxygenases [50]. Of those, laccase, widely used to catalyze the oxidation of certain aromatic substrates (i.e., phenolic compounds), can play a pivotal role in the catalysis process of tetracycline consisting of a phenolic ring and keto-enol bonds [50,51]. Accordingly, many studies have reported the high effectiveness of applying fungi-derived laccases to wastewaters in TC degradation and remediation [51,52,53]. In addition, such an effect of laccase in plants has been reported in a few cases where it has been applied to some herbicides, including atrazine, isoproturon, 2,4,6-trichlorophenol, etc. [54,55]. However, no studies on self-degradation mechanisms in plants for antibiotics have been conducted on crop species. Interestingly, in our study, the effect of hormesis response on *B. campestris* seedlings under low concentrations (5 and 10 mg/L) of TC indicates that the seedlings might potentially adjust the antibiotic exposure by mitigating it to better growth.

In Korea, the livestock population is increasing due to increased meat consumption, which will further promote the introduction of antibiotics, such as tetracycline, into the soil and water environments. In particular, the application of manure fertilizers to agricultural soils, which has become a de facto means of animal waste treatment, is raising concerns about crop safety and productivity due to antibiotic toxicity. Moreover, given the complexity of soils, the presence of additional contaminants, e.g., heavy metals, pesticides, etc., may affect the fate and toxicity of coexisting antibiotics [13,56]. Although this study offers a valuable insight into the phytotoxic effects of TC on cabbage seedlings, it was carried out in a controlled setting to intentionally minimize these external influences, especially, microbial interactions found in natural soil settings. In addition to detoxifying pollutants like antibiotics, the rhizosphere microbiome is essential for hormone synthesis, nutrient cycling, and plant stress responses [57]. Given such rhizobiotic dynamics, future studies should investigate the three-dimensional interactions between plants, microorganisms, and veterinary antibiotics by integrating rhizosphere-inclusive systems, such as soil-based or field environments. These kinds of studies are crucial for developing biologically integrated and sustainable mitigation methods as well as for improving our understanding of the ecological concerns posed by residual antibiotics.

Overall, our findings reveal that tetracycline adversely affected seed germination, early growth, and development of cabbage plants primarily by inhibiting root elongation and photosynthetic functions and provided insight into the antibiotic dynamics mechanism in the crops that are associated with accumulation and degradation. In addition, the outcomes may help to explore the approach of the One health framework, which could promote appropriate antibiotic use, education, and social action. However, these results are based on a mesocosm study performed in controlled conditions, their application in the field will inevitably be limited. Therefore, further studies on the toxicity of TC to crops under field conditions, encompassing a variety of environments and treatments, are essential to ensure food production safety and the sustainability of agroecosystem. Additionally, future research should focus on gaining a deeper understanding of the metabolic processes involved in the degradation of TC in plants. It should also include screening for the distribution of TC in both plant and soil, as this approach shows promise in identifying substances related to TC compounds. That is, by identifying specific TC-degrading substances, the underlying metabolic responses of seedlings to TC stress in plants can be elucidated.

## 4. Materials and Methods

### 4.1. Plant Materials and Chemical Agents

Seeds of cabbage (*Brassica campestris* L. ssp. *Pekinensis* Rupr.) were purchased from the ASIA seed company (Seoul, Republic of Korea) and stored at 4 °C until sowing. For the antibiotic agent, a powder of tetracycline hydrochloride (C_22_H_24_N_2_O_8_∙HCl, purity 98.0%, CAS No. 64-75-5) was purchased from Sigma Aldrich Co. (Yongin, Republic of Korea).

### 4.2. Germination Test

Twelve seeds of cabbage were placed on a Petri dish containing 1.2% water–agar medium. There were five different TC dose treatments, including 0 (control), 5, 10, 25, and 50 mg/L, and each treatment had three replicates. Petri dishes were incubated vertically for a better visualization of root elongation in a growth chamber under constant conditions, with a photoperiodic lighting of 16 h light/8 h dark and a temperature of 20 ± 2 °C. Seed germination was measured every 24 h for three days and its percentage was calculated as follows:

$$Germination\;percentage\;(GP,\;\%)=\frac{Number\;of\;germinated\;seeds}{Total\;number\;of\;seed\;tested}\times 100$$

### 4.3. Pot Experiment

Seeds of *B. campestris* were sown in pots (⌀: 9 cm, height: 10 cm) containing a substrate mixture of coco peat and perlite (artificial soil). Tetracycline treatments were administered at two distinct growth stages of cabbage: initially at sowing and three weeks after sowing (late seedling stage with emergence of fifth leaf). Tetracycline treatments were prepared in high concentration stock (1000 mg/L) then applied in five different doses (0, 5, 10, 25, and 50 mg/L) along with negative control treatments consisting of pots without plants for each dose. Each treatment had three replicates. Cabbage plants were cultivated in a growth chamber (JSPC-960C, JSR, Gongju, Republic of Korea) for six weeks under controlled conditions: 20 ± 2 °C, 60% relative humidity, and a 16 h light/8 h dark photoperiod. Analysis of biometric characteristics for the cabbage plants was conducted biweekly for six weeks.

### 4.4. Analytical

To evaluate the physiological growth of in vitro-grown cabbage seedlings, the FW of shoots, as well as the length of shoots and roots, were measured eight days after sowing. For the pot experiment, morphological and physiological characteristics, including photosynthetic rate, pigments, FW, dry weight (DW), and LN, were assessed biweekly over the six weeks of cultivation. Fresh weight and LN of each plant were recorded directly after each sampling, while DW was measured after freeze-drying at –55 °C for 72 h. To assess the photosynthetic rate, the minimum PSII quantum yield (*F*_v_/*F*_m_) in cabbage leaves was measured using a portable fluorimeter with leaf-clip holders (MINI-PAM-II, Heinz Walz GmbH, Effeltrich, Germany). The *F*_v_/*F*_m_ ratio was calculated as *F*_v_/*F*_m_ = (*F*_m_ − *F*_0_)/*F*_m_; where *F*_0_ is the minimal fluorescence emitted when PSII reaction centers are open, F_m_ is the maximum fluorescence emitted when the reaction centers are close and the variable fluorescence *F*_v_ is the difference between *F*_m_ and *F*_0_. To determine chlorophyll and carotenoid contents in the plants, leaf samples (0.1 g) were extracted with 80% acetone, and the extract was measured at 470, 645, and 663 nm wavelengths using UV-160A spectrophotometer (Shimadzu, Kyoto, Japan). Total chlorophyll and carotenoid contents were calculated using the formula described in Kim et al. [58].

Tetracycline was extracted from soil and cabbage tissues by solid phase extraction (SPE) pretreatment combined with LC-MS/MS (liquid chromatography-tandem mass spectrometry) (API4000, ABSCIEX, Toronto, Canada) analysis following a modified method from Wallace and Aga [59]. Samples collected were lyophilized and then ground into fine powder for homogenization. Samples of soil (0.5 g) and plant (0.1 g) were mixed with 20 mL of McIlvaine buffer (pH 3.6) and 250 μL of 5% Na_2_-EDTA (*w*/*v*) extraction solvents, vortexed for 15 min, ultra-sonicated for 10 min (40 kHz, 120 W), and then centrifuged at 4000 rpm (Combi514R, Hanil Biomed Inc., Seoul, Republic of Korea). The resulting extracts were vacuum-filtered, diluted with 15 mL of HPLC-grade water, and purified with SPE using LEOX^®^ plus Cartridge (3 mL/60 mg, Bekolut, Hauptstuhl, Germanry). Each sample was loaded into a cartridge activated with 3 mL methanol, 3 mL 0.5 M HCl, and 3 mL HPLC-grade water. The collected antibiotic-extract solution was eluted with MeOH and then concentrated to a final volume of 500 μL using a nitrogen concentrator (TS-A0040, SCINCO, Seoul, Republic of Korea) in a constant-temperature water tank at 35 °C. The final antibiotic extract was filtered through a 0.22 μm PTFE (Polytetrafluoroethylene) syringe filter and stored at –20 °C until analysis.

### 4.5. Bioconcentration Factor and Total Accumulation Content

To assess the capacity of plant uptake for antibiotics, the bioconcentration factor (BCF) was calculated with the following formula:

$$BCF=\frac{TC\;concentrationn\;in\;plant}{TC\;concentration\;in\;soil}$$

The values of BCF were obtained from samples in Week 4 and Week 6. Meanwhile, the actual accumulation of TC in the plant (g per plant) was calculated as follows:

$$TC\;accumulation\;in\;plant\;(g/plant)\;=Concentration\;of\;TC\;in\;plant\;\left(mg/L\right)\times DW\;of\;one\;plant\;(g)$$

### 4.6. Statistical Analysis

To investigate significant differences in variables of soil and plant among different TC dose treatments (*n* = 3), a one-way analysis of variance (ANOVA) with Tukey’s honestly significant difference (HSD) post hoc test (*p* < 0.05) was performed. Principal component analysis (PCA) was used to interpret patterns of variation in the dataset obtained from the pot experiments in Week 4 and Week 6, focusing on the response of the parameters to TC dose levels and inter-parameter relationships. Statistical analysis was performed using Minitab software version 16 (Minitab Inc., State College, PA, USA) and the R program version 3.3.3.

## 5. Conclusions

The present study investigated the physiological responses of Kimchi cabbage, a major crop in Korea, to tetracycline toxicity during early growth stages from seed germination to vegetative growth and its accumulation in the plant tissues. According to in vitro test results, seed germination was delayed at high doses of tetracycline (>25 mg/L). Additionally, early growth parameters like shoot and root length displayed dose-dependent reduction, most notably at 50 mg TC/L with root elongation being particularly sensitive. Similarly, in the pot experiment, physiological parameters, e.g., FW, LN, pigment content, and photosynthetic efficiency, showed a significant decrease at 50 mg TC/L. Meanwhile, lower doses of 5 and 10 mg TC/L appeared to have a hormesis effect boosting biomass and the overall morphological phenotype. Immediately after TC exposure, the bioaccumulation factors ranging from 0.5 to 1 suggest that tetracycline accumulated in plant tissues may pose a potential risk to human health if consumed, but biodegradation mechanisms within plants would reduce the risk to less than expected. Although this study confirmed the phytotoxicity and threshold of TC for cabbage, further studies targeting various crop species and large-scale applications should be designed to better monitor the productivity, quality, and safety of crops grown in antibiotic-contaminated field environments, thereby contributing to the sustainability of agricultural ecosystems.

## Figures and Tables

**Table 1:** Temporal change in germination percentage (%) of cabbage seeds (*Brassica campestris* L.) treated with different doses of tetracycline (TC).

Time	Tetracycline Dose (mg/L)				
	0	5	10	25	50
Day 1	91.7 ± 0 ^a^	88.9 ± 4.8 ^a^	88.9 ± 4.8 ^a^	75.0 ± 0 ^b^	69.4 ± 4.8 ^b^
Day 2	100 ± 0 ^a^	94.4 ± 4.8 ^a^	91.7 ± 0 ^a^	94.4 ± 4.8 ^a^	97.2 ± 4.8 ^a^
Day 3	100 ± 0 ^a^	100 ± 0 ^a^	100 ± 0 ^a^	100 ± 0 ^a^	100 ± 0 ^a^

Data are the mean ± standard deviations (*n* = 3). Different letters indicate significant differences among TC dose treatments (Tukey’s HSD, *p* < 0.05).

**Table 2:** Values of photosynthetic rate (the maximum PSII quantum yield; *F*_v_/*F*_m_) and pigments (SPAD, chlorophylls, or carotenoids) in cabbage (*B. campestris* L.) leaves grown under different tetracycline (TC) doses for 6 weeks.

Treatment (mg TC/L)	The Maximum PSII Quantum Yield			Pigment Content				
	*F*_v_/*F*_m_			SPAD	Chlorophylls (mg/g)		Carotenoids (mg/g)	
	Week 2	Week 4	Week 6	Week 2 ^†^	Week 4	Week 6	Week 4	Week 6
0	619.5 ± 14.5 ^a^	794.0 ± 6.0 ^a^	577.0 ± 4.0 ^ab^	20.7 ± 1.7 ^a^	355.5 ± 10.5 ^a^	356.3 ± 0.6 ^a^	49.8 ± 3.2 ^a^	56.3 ± 3.4 ^a^
5	641.5 ± 0.5 ^a^	790.5 ± 7.5 ^a^	610.5 ± 1.5 ^a^	20.6 ± 0.6 ^a^	209.0 ± 0.1 ^d^	318.7 ± 0.3 ^c^	27.4 ± 1.9 ^c^	49.0 ± 0.1 ^b^
10	635.0 ± 8.0 ^a^	743.0 ± 42.0 ^a^	583.0 ± 0.0 ^ab^	20.5 ± 0.3 ^a^	248.0 ± 0.9 ^c^	322.7 ± 0.2 ^b^	35.4 ± 1.0 ^b^	48.6 ± 0.4 ^b^
25	628.5 ± 15.5 ^a^	794.5 ± 12.5 ^a^	583.0 ± 0.0 ^ab^	21.1 ± 1.0 ^a^	261.2 ± 0.6 ^b^	211.3 ± 0.5 ^e^	36.3 ± 0.6 ^b^	35.9 ± 0.2 ^d^
50	644.5 ± 4.5 ^a^	501.0 ± 23.0 ^b^	536.0 ± 47.0 ^b^	19.2 ± 0.3 ^a^	197.5 ± 0.4 ^e^	236.5 ± 0.2 ^d^	25.3 ± 0.4 ^c^	38.8 ± 0.1 ^c^

Data are mean ± standard deviations (*n* = 3). Different letters within each column indicate significant differences among the TC treatments (Turkey’s HSD, *p* < 0.05). ^†^ For relative comparison, pigments were measured using SPAD due to limitations in sample quantity.

**Table 3:** Accumulation amount and bioconcentration factor (BCF) of *B. campestris* seedlings grown in soils treated with different tetracycline (TC) doses.

Treatment (mg TC/L)	BCF		Accumulation Amount (g/plant)	
	Week 4	Week 6	Week 4	Week 6
0	0.091 ± 0.030 ^c^	0.213 ± 0.110 ^b^	0.003 ± <0.001 ^d^	0.003 ± 0.001 ^d^
5	0.458 ± 0.011 ^b^	0.636 ± 0.037 ^a^	1.638 ± 0.344 ^cd^	0.834 ± 0.155 ^c^
10	0.610 ± 0.093 ^ab^	0.205 ± 0.091 ^b^	5.623 ± 0.119 ^c^	0.862 ± 0.225 ^c^
25	0.780 ± 0.023 ^a^	0.159 ± 0.018 ^b^	23.980 ± 1.677 ^b^	3.307 ± 0.578 ^b^
50	0.424 ± 0.136 ^b^	0.066 ± 0.021 ^b^	31.485 ± 3.735 ^a^	4.179 ± 0.062 ^a^

Data are mean ± standard deviations (*n* = 3). Different letters indicate significant differences among the TC treatments (Tukey’s HSD, *p* < 0.05).

## Data Availability

The datasets used during the current study are available from the corresponding author upon reasonable request.

## Abbreviations

The following abbreviations are used in this manuscript:

TC	Tetracycline
GP	Germination percentage
BCF	Bio-concentration factor
VAs	Veterinary antibiotics
FW	Fresh weight
DW	Dry weight
LN	Leaf number
*F*_v_/*F*_m_	The maximum photosystem II quantum yield
SPE	Solid phase extraction
PS	Photosystem
